# Impact of Corticosteroids on the Proportions of Circulating Tfh Cell Subsets in Patients With Systemic Lupus Erythematous

**DOI:** 10.3389/fmed.2022.949334

**Published:** 2022-07-05

**Authors:** Minjing Mao, Shuqin Xu, Lin Lin, Danfeng Dong, Minghui Xue, Siwei He, Gang Cai

**Affiliations:** ^1^Department of Laboratory Medicine, Ruijin Hospital, Shanghai Jiao Tong University Medical School, Shanghai, China; ^2^The Cancer Hospital of the University of Chinese Academy of Sciences (Zhejiang Cancer Hospital), Institute of Basic Medicine and Cancer, Chinese Academy of Sciences, Hangzhou, China

**Keywords:** T follicular helper cell, corticosteroid, immunoregulation, immune cells, systemic lupus erythematosus

## Abstract

**Objectives:**

This study aimed to analyze the distribution of T follicular helper (Tfh) cells in lupus patients, and the effects of steroids on circulating Tfh cells.

**Methods:**

Circulating Tfh cell subsets were defined by multicolor flow cytometry as Tfh17, Tfh2 or Tfh1 subpopulations of CXCR5^+^CD45RA^–^CD4^+^ T cells in the peripheral blood of SLE patients and healthy controls. To test the effects of corticosteroid on Tfh cells, PBMC harvested from both SLE and healthy controls were cocultured with dexamethasone, and then analyzed by Flow cytometry.

**Results:**

The proportion of Tfh17 cells in SLE patients was increased significantly compared with healthy controls. Additionally, patients with an active disease had reduced Tfh1 subsets than those with an inactive disease and healthy controls. The frequency of Tfh2 cells was associated with the proportion of circulating plasmablasts and the amount of anti-dsDNA. Dexamethasone reduced the percentage of Tfh2 cells while increased the proportion of Tfh17 subset in gated CXCR5^+^CD45RA^–^CD4^+^ T cells.

**Conclusion:**

Our study investigated the distribution of circulating Tfh subsets in lupus patients. Corticosteroids treatment not only down-regulated the proportion of circulating Tfh cells, but also altered the distribution of Tfh subsets *in vivo* and *in vitro*.

## Introduction

It has been widely described that the interaction between CD4^+^ T cell and B cell promotes antibody production in the germinal centers (GCs) of secondary lymphoid organs, the sites where Ig isotype switching and affinity maturation occur, subsequently giving rise to memory B cells and long-lived plasma cells (1). Pathogenic autoantibodies produced by autoreactive memory B cells upon restimulation undergo class switching and somatic mutation to achieve affinity maturation, and these features are consistent with GC selection. System lupus erythematosus (SLE) is a prototype autoimmune disease characterized by the production of multiple autoantibodies, particularly antinuclear antibodies (2). The involvement of aberrant GC responses in autoantibody production finds support by the evidence of exuberant GC activity in patients with active lupus nephritis, as well as by observations of spontaneous GC formation in murine lupus (3, 4). These findings indicate that GC is the site where autoreactive B cell maturation occurs in SLE.

T follicular helper (Tfh) cells are the primary cells regulating T cell-dependent B cell maturation in GCs (5). Tfh cells express the chemokine receptor CXCR5, which enables them to migrate along the CXCL13 gradient into B cell follicles (6). Additionally, inducible costimulator (ICOS) and programmed death-1 (PD-1) are demonstrated to be highly expressed on the surface of Tfh cells (7, 8). ICOS mediates the development and expansion of Tfh cells by interacting with dendritic cells or B cells, while PD-1 signaling limits the number of Tfh cells and enhances their ability to upregulate the expression of interleukin (IL)-21, which plays an important role in B cell selection and survival (9–11). Thus, abnormal Tfh cell responses may contribute to the pathogenesis of SLE.

Studies regarding the regulatory role of Tfh cells in human SLE are limited, probably because Tfh cells reside in the GCs of secondary lymphoid organs, which renders routine sampling difficult. However, a subset of CXCR5^+^CD4^+^ T cells (CD45RA^–^ or CD45RO^+^) within the memory cell compartment was shown to drive *in vitro* differentiation of naïve B cells and memory B cells into Ig-secreting PCs (12). Additionally, a PD-1^+^ subset of CXCR5^+^CD4^+^ T cells in blood was expanded in some patients with severe SLE, and was positively correlated with autoantibody titers (13). These findings suggest that circulating CXCR5^+^CD4^+^ memory T cells represent a Tfh cell counterpart, reflecting the responses of GC. However, the information about circulating Tfh cells in SLE patients is still limited now. The relationship between circulating Tfh cells and disease activity and aberrant B cell responses remains controversial, partially due to using different immunoregulatory regimens including steroids. Moreover, based on CCR6 and CXCR3 expression levels, circulating Tfh cells can be classified into three subclasses, including Tfh1 (CCR6^–^CXCR3^+^), Tfh2 (CCR6^–^CXCR3^–^) and Tfh17 (CCR6^+^CXCR3^–^) (14). Since the imbalance between Tfh subclasses has been reported to contribute to disease activity, it is necessary to understand whether steroid use influence this balance.

In this study, we found that the frequency of circulating Tfh cells in blood from patients with SLE was comparable with that in healthy donors, but the distribution of circulating Tfh cell subsets was dramatically different between patients and healthy controls. Steroid use decreased the number of Tfh cells, but Tfh cell seemed to be hyporesponsive after prolonged stimulation with steroids. Meanwhile, steroid use increased the proportion of Tfh17 cells and decreased the proportion of Tfh2 and Tfh1 cells both *in vitro* and *in vivo*.

## Materials and Methods

### Patients and Healthy Individuals

A total of 27 patients who were newly diagnosed with SLE and 20 healthy controls were enrolled. Clinical characteristics of patients and healthy controls were presented in Table 1. The diagnosis of patients met the American College of Rheumatology classification criteria for SLE (15). Disease activity was assessed by the SLE disease activity index (SLEDAI) (16). Patients who were treated with biological regimens or cytotoxic drugs were excluded in our study. Because it was reported that low dosage of steroids (<20 mg/d) did not have impact on the frequency of circulating Tfh cells (17), patients who received more than 20 mg of steroids per day were also excluded. To evaluate the influence of secondary diseases, patients with a disease duration longer than half a year were also excluded. The level of anti-nuclear Abs (ANAs) was determined by indirect immunofluorescence labeling of Hep-2 cells. Anti-dsDNAs were detected by ELISA (Bio-Rad Lab. Inc., CA, United States). Written informed consent was obtained from each participant in agreement with the Helsinki declaration, under which the study was reviewed and approved by the Ethics Committee of Ruijin Hospital, Shanghai Jiao Tong University School of Medicine.

**TABLE 1:** Clinical characteristics of patients and healthy controls.

Characteristics		SLE patients (*n* = 27)	Healthy controls (*n* = 20)
Gender	Male	2	3
	Female	25	17
Age (mean ± SEM)		41 ± 16	37 ± 13
SLEDAI	≤4	14	
	>4	13	
Anti-dsDNA	+	17	
	–	10	

*A total of 27 patients who were newly diagnosed with SLE and 20 healthy controls were enrolled.*

*The diagnosis of patients met the American College of Rheumatology classification criteria for SLE.*

### Flow Cytometry Analysis

We isolated peripheral blood mononuclear cells (PBMCs) from the blood samples using density-gradient centrifugation on Ficoll-Paque. Single-cell suspensions were treated with fixable viability stain 510 (BD Bioscience, 564406) for half an hour, followed by staining with the following antibodies to distinguish the Tfh subsets: FITC- conjugated anti-CD45RA (BioLegend, N418), PE-conjugated anti-CCR6 (BioLegend, G034E3), PerCP/Cy5.5-conjugated anti-CXCR5 (BioLegend, J252D4), PE/Cy7-conjugated anti-CXCR3 (BioLegend, G025H7), APC-conjugated anti-PD-1 (BioLegend, EH12.2H7), and APC/Cy7- conjugated anti-CD4 (BioLegend, RPA-T4). For the detection of circulating plasma cells, PBMCs were stained with FITC-conjugated CD27 (BioLegend, M-T271), PE-conjugated IgD (BioLegend, W18340F), PerCP/Cy5.5-conjugated anti-CD19 (BioLegend, HIB19), and APC-conjugated CD38 (BioLegend, HB-7), followed by the treatment with fixable viability stain 510. Cell acquisition was performed using a CantoII cytometer (BD Bioscience). Data were analyzed with Diva (BD) software.

### Cell Culture

PBMCs were collected by Ficoll-Paque Plus density gradient centrifugation and resuspended in RPMI 1640 medium containing 50 μM 2-mercaptoethanol, 1 mM Sodium Pyruvate, 50 mg/mL streptomycin, 50 U/mL penicillin, and 10% heat inactivated FCS. Then Cells were seeded into a 24-well plate (Corning, Tewksbury, MA, United States) at 1.0 × 10^6^ per well, incubated at 37°C in a humidified atmosphere with 5% CO2 for 24 h, and were subjected to FACS analysis. To observe the effect of dexamethasone on Tfh subsets, PBMCs cultured in RPMI 1640 medium were treated with 10 ng/mL IL-6 (Solarbio, China) in the presence or absence of dexamethasone (1 mg/mL, Pfizer manufactory, Belgium, NV, United States). Experiments were carried out in duplicates with equal cell number among comparable groups. After 24 and 72 h, cells were harvested and then stained with antibodies for the detection of Tfh subsets.

### Statistical Analysis

All data were presented as mean ± standard error of the mean (SEM). One-way analysis of variance (ANOVA) and student’s 2-tailed *t*-test was used to compare the differences between groups. Alterations of Tfh subsets before and after dexamethasone treatment were analyzed by paired *t*-test. For correlation analyses, Spearman’s correlation coefficients or Pearson’s correlation coefficients with a 2-tailed *P* value were determined. A *P* value less than 0.05 was considered significant difference. Statistical analysis was with Prism software (version 6.0; GraphPad Software).

## Results

### Circulating CD4^+^CD45RA^–^CXCR5^+^ Cells in System Lupus Erythematosus Patients

**FIGURE 1:**
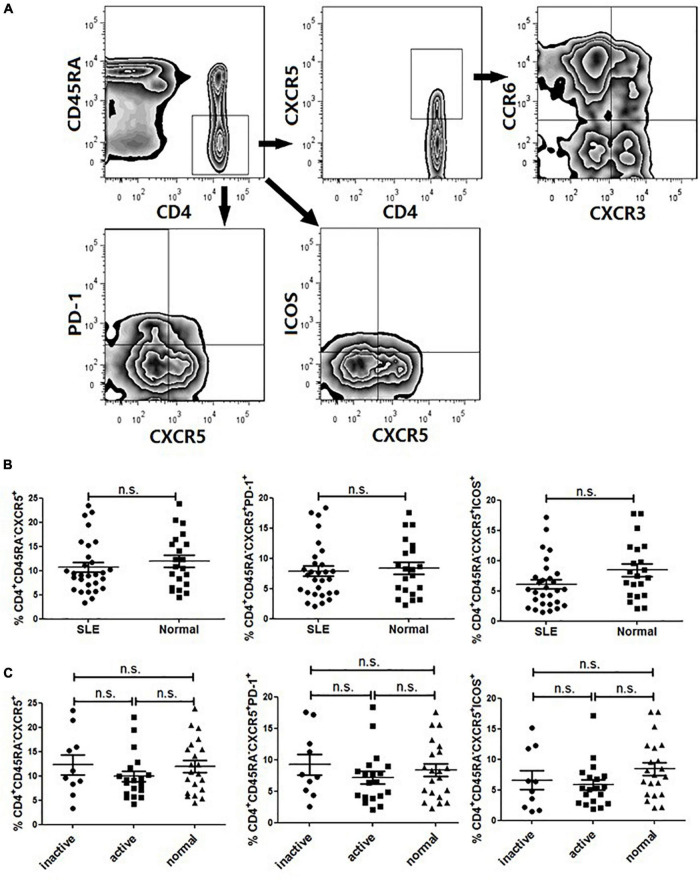
FACS analysis and circulating Tfh cells in SLE patients. PBMCs were isolated and stained with labeled antibodies specific for CD4, CD45RA, CXCR5, ICOS, PD-1, CCR6, CXCR3. **(A)** Circulating Tfh cells were gated in CD4^+^CD45RA^–^CXCR5^+^, CD4^+^CD45RA^–^PD-1^+^ and CD4+CD45RA-ICOS+ respectively. Then Tfh subsets were determined by CXCR3 and CCR6 expression on gated CD4^+^CD45RA^–^CXCR5^+^ T cells, allowing the identification of Tfh1 (CXCR3^+^CCR6^–^), Tfh2 (CXCR3^–^CCR6^–^) and Tfh17 (CXCR3^–^CCR6^–^) subsets. **(B)** Frequencies of CD4^+^CD45RA^–^CXCR5^+^, CD4^+^CD45RA^–^PD-1^+^ and CD4^+^CD45RA^–^ICOS^+^ cell subsets were compared between SLE patients and healthy controls. **(C)** Frequencies of CD4^+^CD45RA^–^CXCR5^+^, CD4^+^CD45RA^–^PD-1^+^ and CD4^+^CD45RA^–^ICOS^+^ cell subsets were compared between inactive SLE patients, active SLE patients and healthy controls. All results are presented as mean ± standard error of the mean (SEM). ns: not significant.

As several studies suggested that circulating Tfh cells were associated with autoimmunity, we first compared the frequency of CD4^+^CD45RA^–^CXCR5^+^ cells in total CD4^+^ T cells in patients with SLE (14 with a SLEDAI ≤ 4 and 13 with a SLEDAI > 4) with that in healthy controls (20 age- and sex-matched). Patients were given different dosage of steroids (<20 mg/day), and they did not receive any biological or cytotoxic treatments in this study. The gating strategy was used to identify circulating Tfh cells by flow cytometry, as represented in Figure 1A.

The frequency of CXCR5^+^ cell subset in memory CD4^+^ T cells (CD4^+^CD45RA^–^) was not substantially different between SLE patients and healthy controls (Figure 1B). Since PD-1 and ICOS were typical surface molecule markers of Tfh cells in GCs, we also analyzed the gated PD-1^+^- or ICOS^+^CXCR5^+^-expressing CD4^+^CD45RA^–^ cells (Figure 1A). The results showed that there was no significant differences between SLE patients and healthy controls (Figure 1B) (17). Then, we compared the frequency of circulating Tfh cells between active patients (SLEDAI > 4) and inactive patients (SLEDAI ≤ 4). It seemed that the frequency of circulating PD-1^+^ Tfh cells in active patients was higher than that in inactive patients, but the difference did not reach statistical significance (Figure 1C).

### Abnormal Distribution of Tfh Subsets in Active System Lupus Erythematosus Patients

**FIGURE 2:**
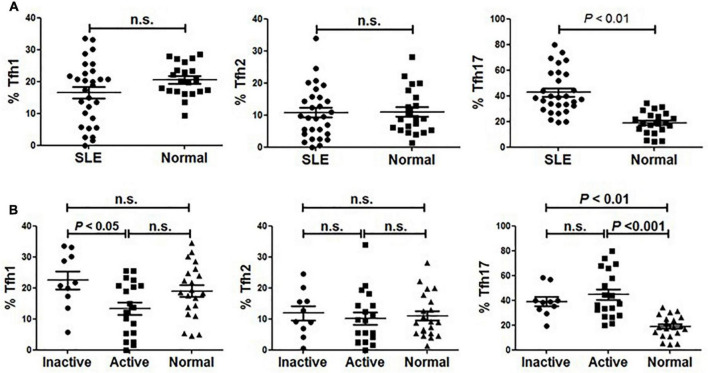
Abnormal distribution of Tfh1 and Tfh17 cells in active SLE patients. **(A)** Frequencies of Tfh1, Tfh2 and Tfh17 cells were analyzed and compared between SLE patients and healthy controls. **(B)** Frequencies of Tfh1, Tfh2 and Tfh17 cells were analyzed and compared between inactive SLE patients, inactive SLE patients and healthy controls. All results are presented as mean ± standard error of the mean (SEM). n.s., not significant.

As described by Morita and colleagues, different expression combinations of chemokine receptor CXCR3 and CCR6 defined three major subsets within blood circulating CD4^+^CXCR5^+^ T memory cells, including Tfh1 (CXCR3^+^CCR6^–^), Tfh2 (CXCR3^–^CCR6^–^) and Tfh17 (CXCR3^–^CCR6^+^) cells (14). We analyzed these subsets distribution in our cohort comprising SLE patients and age- and sex-matched healthy individuals. As shown in Figure 2, the frequency of Tfh17 cells within CD4^+^CD45RA^–^CXCR5^+^ cells in SLE patients was significantly higher than that in healthy individuals, while neither Tfh1 nor Tfh2 frequency was found to be significantly decreased in SLE patients (Figure 2A).

To determine whether Tfh cell subsets were associated with disease activity, we then compared the frequency of each subset between active and inactive patients. We found that the frequency of Tfh1 cells in active SLE patients was significantly lower than that in inactive patients or healthy controls (Figure 2B). Meanwhile, patients with active disease had a significantly higher frequency of Tfh17 cells than normal healthy controls (Figure 2B). However, no significant difference in Tfh2 cell frequency was found when comparing active and inactive patients and healthy controls (Figure 2B).

### Abnormal Distribution of Circulating Tfh Subsets Was Associated With Humoral Responses of System Lupus Erythematosus

**FIGURE 3:**
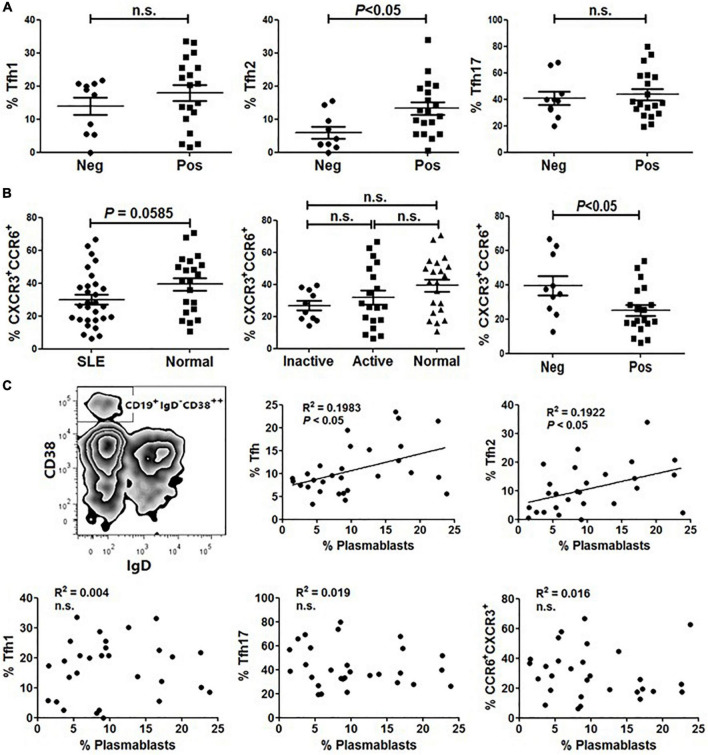
The frequency of Tfh2 cells is significantly higher in ANA positive SLE patients and positively correlated with plasmablasts. **(A)** Frequencies of Tfh1, Tfh2 and Tfh17 cells were analyzed and compared between anti-dsDNA negative SLE patients and anti-dsDNA positive SLE patients. **(B)** The frequency of CXCR3^+^CCR6^+^ subset on gated CD4^+^CD45RA^–^ T cells was analyzed and compared between SLE patients (including inactive and active SLE patients, anti-dsDNA negative SLE patients and anti-dsDNA positive SLE patients) and healthy controls. **(C)** Plasmablasts were gated as CD19^+^IgD^–^CD38^++^ population, and the correlations between the amount of plasmablasts and frequencies of each type of Tfh cells and Tfh subsets from SLE patients were analyzed. All results are presented as mean ± standard error of the mean (SEM). n.s., not significant.

Tfh cells play a critical role in the development of Ag-specific humoral responses and anti-dsDNA autoantibodies are considered a specific marker for lupus (18, 19). We then investigated the relationship between anti-dsDNA autoantibodies and Tfh cell subset distribution in SLE. As shown in Figure 3A, the frequency of Tfh2 cells in patients harboring anti-dsDNA autoantibodies was significantly higher than that in patients without anti-dsDNA antibodies (Figure 3A). However, neither Tfh1 nor Tfh17 showed a difference between the two groups (Figure 3A). Besides, it was interesting to see a significantly reduced proportion of CCR6^+^CXCR3^+^ cells in patients having anti-dsDNA autoantibodies (Figure 3B). We also assessed whether CD19^+^IgD^–^CD38^++^ plasmablasts (Figure 3C) in SLE patients were correlated with the proportion of Tfh subsets. The results indicated that the proportion of circulating Tfh and Tfh2 subsets was positively correlated with the level of plasmablasts, respectively (Figure 3C).

### The Impact of Dexamethasone on Circulating Tfh Cells in Patients Receiving Steroid Impulse

**FIGURE 4:**
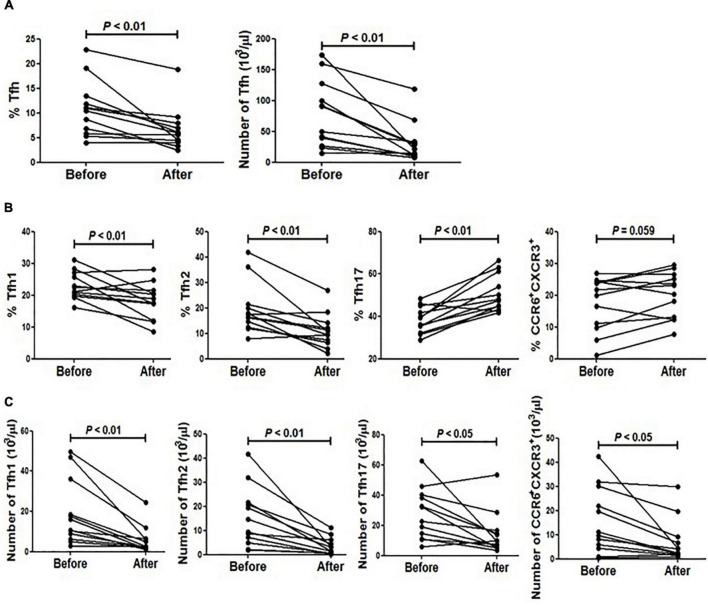
The impact of methylprednisolone on circulating Tfh cells from SLE patients. **(A)** The frequency and absolute number of Tfh cells before and after methylprednisolone treatment were analyzed. **(B)** Frequencies of all Tfh subsets before and after methylprednisolone treatment were analyzed. **(C)** Absolute number of all Tfh subsets before and after methylprednisolone treatment was analyzed. All results are presented as mean ± standard error of the mean (SEM).

Since all included patients in this study were treated with different doses of steroid (10–20 mg of dexamethasone per day), we wondered whether the corticosteroids had an effect on circulating Tfh cells. To clarify this point, we randomly selected 12 patients who were intravenously injected with high doses of methylprednisolone (100–400 mg per day for 3–4 days), then compared the frequency of circulating Tfh cells in these patients before and after steroid impulse. As shown in Figure 4, there was a significant reduction of circulating Tfh cells after steroid treatment (Figure 4A). To exclude the influence of corticosteroids on total lymphocyte number, the absolute numbers of pre- and post-treatment Tfh cells were measured. Our data showed that the absolute number of Tfh cells was also decreased after steroid treatment (Figure 4A).

We then analyzed the distribution of circulating Tfh cell subsets upon methylprednisolone impulse. The results showed that the frequency of Tfh17 was increased while that of Tfh2 and Tfh1 was decreased (Figure 4B). Additionally, CXCR3^+^ and CCR6^+^ double positive cells were also increased significantly (Figure 4B). However, as the absolute number of lymphocytes was decreased after methylprednisolone treatment, the frequencies of all Tfh subsets were decreased (Figure 4C).

### Circulating Tfh Cells in System Lupus Erythematosus Patients Were Resistant to Methylprednisolone

**FIGURE 5:**
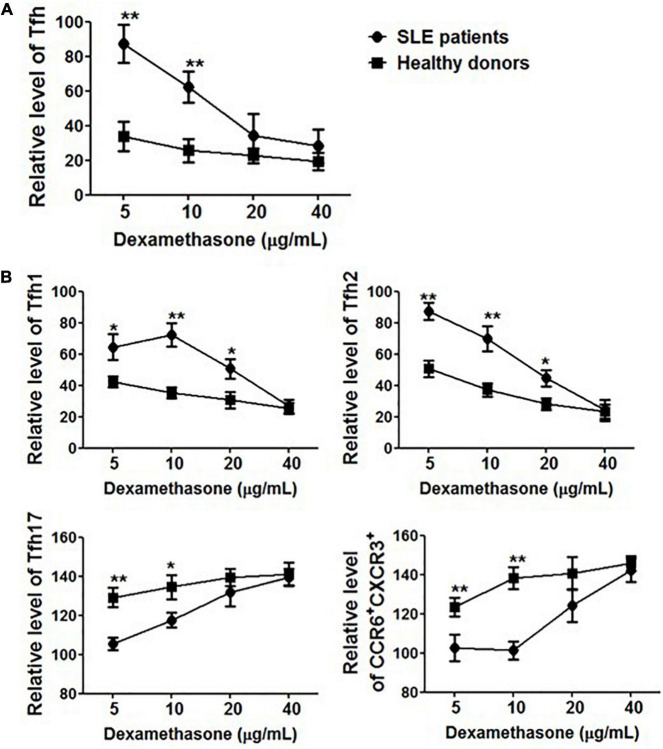
The effect of methylprednisolone on Tfh cells. **(A)** The relative frequencies of Tfh cells from both SLE patients and healthy controls were measured 24 h after treatments with methylprednisolone at different dosages (5 μg/ml, 10 μg/ml, 20 μg/ml, and 40 μg/ml). **(B)** The relative frequencies of Tfh subsets from both SLE patients and healthy controls were measured 24 h after treatments with different dosages of methylprednisolone (5 μg/ml, 10 μg/ml, 20 μg/ml, and 40 μg/ml). All results are presented as mean ± standard error of the mean (SEM). *p < 0.05, **p < 0.01.

To further elucidate the role of corticosteroids, PBMCs were collected from healthy controls (*n* = 5) and SLE patients (*n* = 5) in the presence of methylprednisolone and cultured *in vitro*. After 24 h, the frequency of Tfh cells and polarization of Tfh subsets were evaluated. Methylprednisolone treatment dramatically decreased the percentage of CD4^+^CD45RA^–^CXCR5^+^ cells in healthy controls, and 5 mg/mL of methylprednisolone was enough to decrease more than 60% of Tfh cells (Figure 5A). However, surprisingly, it seemed that circulating Tfh cells in SLE patients were resistant to methylprednisolone, and 5 mg/mL of methylprednisolone barely down-regulated the frequency of CD4^+^CD45RA^–^CXCR5^+^PD-1^+^ cells. When the concentration of methylprednisolone was increased to 40 mg/mL (Figure 5A), Tfh cell frequency was reduced by 70–80%, which was consistent with our finding on patients who received high-dose methylprednisolone impulse. Tfh subsets also showed an alteration, the proportion of Tfh1 and Tfh2 cells was decreased, while that of Tfh17 and CCR6^+^CXCR3^+^ cells was increased (Figure 5B).

### Methylprednisolone Transiently Regulated the Frequency of Circulating Tfh Cells

**FIGURE 6:**
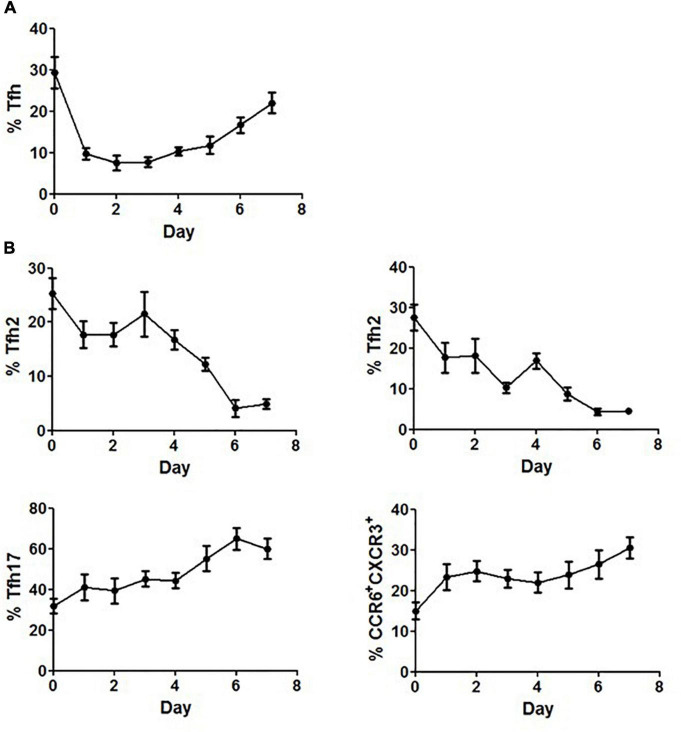
Restoration of Tfh cells after prolonged stimulation with methylprednisolone. **(A)** Circulating Tfh cells from healthy controls were measured daily for up to 7 days after adding 20 mg/mL of methylprednisolone. **(B)** Circulating Tfh subsets from healthy controls were measured daily for up to 7 days after adding 20 mg/mL of methylprednisolone. All results are presented as mean ± standard error of the mean (SEM).

The differential responses of PBMCs to corticosteroids between SLE patients and healthy controls prompted us to further elucidate the effects of corticosteroids on Tfh cells. Freshly isolated PBMCs from healthy controls were cultured in the presence of 20 mg/mL of methylprednisolone for 7 days, and the inhibitory effects of methylprednisolone on CD4^+^CD45RA^–^CXCR5^+^ cells were found to fluctuate with the stimulation time. The frequency of CD4^+^CD45RA^–^CXCR5^+^ cells decreased rapidly at day 1, restored at day 4, and eventually reached a relatively high level at day 7 (Figure 6A). Regarding the distribution of Tfh subsets, we found that the frequency of Tfh1 and Tfh2 cells was continually decreased to the lowest point, while that of Tfh17 and CCR6^+^CXCR3^+^ cells was increased to the highest point during the course of stimulation (Figure 6B).

### Change of Tfh Cells in System Lupus Erythematosus Patients Receiving Corticosteroid Therapy

**FIGURE 7:**
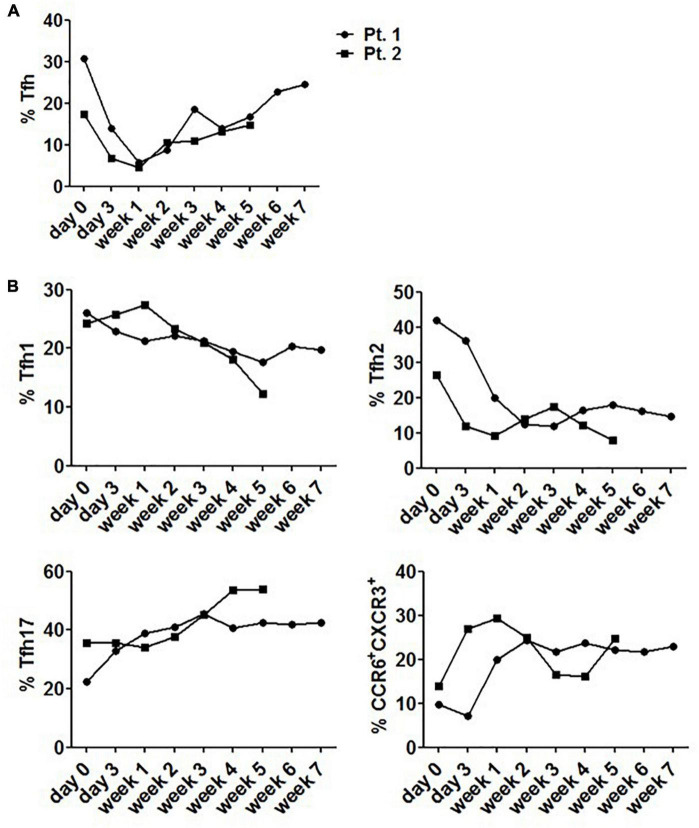
Changes of circulating Tfh cells in SLE patients receiving corticosteroid therapy. **(A)** The level of circulating Tfh cells from 2 newly diagnosed SLE patients fluctuated with 6–8 week steroid therapy period. **(B)** The level of circulating Tfh subsets from 2 newly diagnosed SLE patients fluctuated with 6–8 week steroid therapy period. All results are presented as mean ± standard error of the mean (SEM).

Considering the fluctuation of Tfh cell frequency upon stimulation with corticosteroids, circulating Tfh cells from 2 patients were evaluated 6–8 weeks following steroid therapy. These patients had never received steroid therapy in the past year prior to diagnosis. We found that steroid use rapidly but transiently reduced the frequency of circulating Tfh cells, reaching a nadir at 1 week. After that, the frequency rose and reached a plateau (Figure 7A). We also found that the proportion of Tfh17 and CCR6^+^CXCR3^+^ cells was increased to a plateau during the stable phase of diseases (Figure 7B), while the frequencies of Tfh1 and Tfh2 cells were lower than those before steroid treatment, and remained steady during stable disease phase (Figure 7B).

## Discussion

Tfh cells are crucial immune regulators and their expansion has been demonstrated in murine lupus, which is linked with heightened GC responses and end-organ damage (20, 21). Patients with SLE also have altered GC homeostasis, suggesting that Tfh cells are likewise aberrantly regulated in human diseases. However, it remains controversial about the alteration of peripheral cells bearing a Tfh phenotype. Some research teams reported higher level of Tfh cells in peripheral blood of SLE patients compared with healthy controls (13, 17), while others did not find significant difference in circulating Tfh cells between SLE patients and healthy people (22). The discrepancy among papers may be related to the enrollment of different populations as research subjects and the use of different cell markers to identify circulating Tfh cells. Additionally, the diverse phenotypes and complex pathogenesis of SLE may also explain inconsistent findings. Since the results were not consistent across all previous studies, we aimed to reassess the circulating Tfh cells in SLE patients in this study.

According to the inclusion criteria, 27 SLE patients and 20 healthy controls were finally selected, and the proportion of Tfh cells in peripheral blood was compared between the two groups. The frequency of circulating Tfh cells was not significantly different between SLE patients and healthy controls, neither total CD4^+^CD45RA^–^CXCR5^+^ cells nor gated CD4^+^CD45RA^–^CXCR5^+^ cells expressing PD-1 or ICOS. Additionally, there were no substantial differences between active and inactive patients, indicating that the frequency of Tfh cells was not correlated with disease activity. Our data were consistent with the findings by Carole et al. (22), but were different from another publication by Feng and colleagues (17), which also enrolled Chinese patients as study subjects. They showed that the mean disease duration of included patients was about 7 years, with the longest time exceeding 33 years. Long disease duration of SLE will lead to complex alterations in multiple organs and tissues, and even induce secondary diseases (23). Concerning that the changes caused by secondary diseases might cover up the primary phenotypes of SLE, we only selected patients with a relatively short disease duration (<6 months) as study subjects. Concomitantly, the mean age of our patients was also shorter than that reported in some previous studies.

According to the descriptions by Morita and colleagues, the differential expressions of chemokine receptors CXCR3 and CCR6 were analyzed on CD4^+^CXCR5^+^CD45RA^–^ circulating T cells, which were gated to identify specific Tfh subsets (12). Consistent with the previous report that Tfh17 cells was correlated with the activity of autoimmune diseases, we found that the frequency of Tfh17 cell subset in patients with SLE was significantly higher than that in healthy controls. Additionally, the frequency of Tfh1 cell subset was significantly lower in active SLE patients compared with healthy controls and patients with inactive diseases. Since CXCR3 was involved in T cell migration into inflamed organs, we proposed that the decrease in circulating Tfh1 cell proportion in patients with active SLE was at least partially caused by the accumulation of CXCR3-bearing cells in inflamed organs.

It has been widely accepted that the presence of anti-dsDNA and increased antibody-producing cell population in peripheral blood are typical lupus-related biological indicators (24, 25). Patients with an active disease, especially those with severe SLE, always have a relatively high level of serum anti-dsDNA (26, 27). Since Tfh2 cells were considered to be closely related to the production of IgG and IgE antibodies (12), we explored whether Tfh2 cells were associated with SLE disease process. Our findings indicated that the proportion of Tfh2 subset was increased in patients with anti-dsDNA antibodies, and was positively correlated with the frequency of circulating plasmablasts. However, no significant difference in Tfh2 cell frequency was found between active and inactive patients and healthy controls. Since patients enrolled in this study were treated with low dosage of steroids, we speculated that the intake of steroids might be able to change the proportion of Tfh2 cells, but could not continuously ameliorate the clinical symptoms evaluated by SLEDAI score, leading to the failure to detect the correlation between Tfh2 cell frequency and the course of the disease.

Previous studies have reported that the expansion of Tfh cells in peripheral blood is a normal physiological phenomenon and not caused by immunosuppressive therapy (28, 29). However, our data showed that corticosteroid use could decrease the frequency of circulating Tfh cells in a dose-dependent manner, both *in vivo* and *ex vivo*. Besides, the circulating Tfh cells in SLE patients were more resistant to methylprednisolone treatment compared to those in healthy controls. The possible elucidation was that T cells from SLE patients might be hyporesponsive, as they were previously exposed to *in vivo* stimuli. This explanation was supported by our results that sustained stimulation would reduce the effect of steroids on Tfh cells, both in patients and healthy controls.

Although the frequency of circulating Tfh cells fluctuated upon stimulation with corticosteroids, the Tfh17/Tfh2 ratio or Tfh1 frequency increased continuously. Both *in vivo* and *in vitro* data demonstrated that the proportion of Tfh2 and Tfh1 cells decreased upon steroid stimulation, while that of Tfh17 and CCR6^+^CXCR3^+^ populations increased. To confirm these findings, two patients without receiving steroid treatment in the last six months were involved as controls. During 5–7 weeks of steroid therapy, the changes in Tfh subsets were similar to our finding in *ex vivo* experiments. These data comprehensively demonstrated a modulatory role of steroids on the distribution of circulating Tfh subsets.

Taken together, in this study, we examined the dynamics of circulating Tfh cells in Chinese patients with SLE, and demonstrated that steroids might altered the balance of Tfh cell subsets in peripheral blood. Although many characteristics of circulating Tfh cells have been described, their detailed features and exact roles in SLE development are still unclear. In future studies, we need to enroll patients more strictly and exclude potential confounding factors.

## Data Availability Statement

The original contributions presented in this study are included in the article/supplementary material, further inquiries can be directed to the corresponding author/s.

## Ethics Statement

The studies involving human participants were reviewed and approved by the Ethics Committee of the Ruijin Hospital, Shanghai Jiao Tong University School of Medicine. The patients/participants provided their written informed consent to participate in this study.

## Author Contributions

All authors listed have made a substantial, direct, and intellectual contribution to the work, and approved it for publication.

## Conflict of Interest

The authors declare that the research was conducted in the absence of any commercial or financial relationships that could be construed as a potential conflict of interest.

## Publisher’s Note

All claims expressed in this article are solely those of the authors and do not necessarily represent those of their affiliated organizations, or those of the publisher, the editors and the reviewers. Any product that may be evaluated in this article, or claim that may be made by its manufacturer, is not guaranteed or endorsed by the publisher.

## References

[1] Deenick EK Ma CS. The regulation and role of T follicular helper cells in immunity. *Immunology.* (2011) 134:361–7. 10.1111/j.1365-2567.2011.03487.x 22043829 PMC3230790

[2] Gurevitz SL Snyder JA Wessel EK Frey J Williamson BA. Systemic lupus erythematosus: a review of the disease and treatment options. *Consult Pharm.* (2013) 28:110–21. 10.4140/TCP.n.2013.110 23395811

[3] Luzina IG Atamas SP Storrer CE da Silva LC Kelsoe G Papadimitriou JC Spontaneous formation of germinal centers in autoimmune mice. *J Leukoc Biol.* (2001) 70:578–84. 11590194

[4] Cappione A III Anolik JH Pugh-Bernard A Barnard J Dutcher P Silverman G Germinal center exclusion of autoreactive B cells is defective in human systemic lupus erythematosus. *J Clin Invest.* (2005) 115:3205–16. 10.1172/JCI24179 16211091 PMC1242189

[5] Linterman MA Rigby RJ Wong RK Yu D Brink R Cannons JL Follicular helper T cells are required for systemic autoimmunity. *J Exp Med.* (2009) 206:561–76. 10.1084/jem.20081886 19221396 PMC2699132

[6] Breitfeld D Ohl L Kremmer E Ellwart J Sallusto F Lipp M Follicular B helper T cells express Cxc chemokine receptor 5, localize to B cell follicles, and support immunoglobulin production. *J Exp Med.* (2000) 192:1545–52. 10.1084/jem.192.11.1545 11104797 PMC2193094

[7] Good-Jacobson KL Szumilas CG Chen L Sharpe AH Tomayko MM Shlomchik MJ. PD-1 regulates germinal center B cell survival and the formation and affinity of long-lived plasma cells. *Nat Immunol.* (2010) 11:535–42. 10.1038/ni.1877 20453843 PMC2874069

[8] Xie J Cui D Liu Y Jin J Tong H Wang L Changes in follicular helper T cells in idiopathic thrombocytopenic purpura patients. *Int J Bio Sci.* (2015) 11:220–9. 10.7150/ijbs.10178 25561904 PMC4279097

[9] Park HJ Kim DH Lim SH Kim WJ Youn J Choi YS Insights into the role of follicular helper T cells in autoimmunity. *Immune Netw.* (2014) 14:21–9. 10.4110/in.2014.14.1.21 24605077 PMC3942504

[10] Zhang X Lindwall E Gauthier C Lyman J Spencer N Alarakhia A Circulating CXCR5+CD4+helper T cells in systemic lupus erythematosus patients share phenotypic properties with germinal center follicular helper T cells and promote antibody production. *Lupus.* (2015) 24:909–17. 10.1177/0961203314567750 25654980

[11] Gensous N Schmitt N Richez C Ueno H Blanco P. T follicular helper cells, interleukin-21 and systemic lupus erythematosus. *Rheumatology (Oxford).* (2017) 56:516–23. 10.1093/rheumatology/kew297 27498357

[12] Morita R Schmitt N Bentebibel SE Ranganathan R Bourdery L Zurawski G Human blood CXCR5(+)CD4(+) T cells are counterparts of T follicular cells and contain specific subsets that differentially support antibody secretion. *Immunity.* (2011) 34:108–21. 10.1016/j.immuni.2010.12.012 21215658 PMC3046815

[13] Choi JY Ho JH Pasoto SG Bunin V Kim ST Carrasco S Circulating follicular helper-like T cells in systemic lupus erythematosus: association with disease activity. *Arthritis Rheumatol.* (2015) 67:988–99. 10.1002/art.39020 25581113 PMC4450082

[14] Schmitt N Bentebibel SE Ueno H. Phenotype and functions of memory Tfh cells in human blood. *Trends Immunol.* (2014) 35:436–42. 10.1016/j.it.2014.06.002 24998903 PMC4152409

[15] Smith EL Shmerling R. The American college of rheumatology criteria for the classification of systemic lupus erythematosus: strengths, weaknesses, and opportunities for improvement. *Lupus.* (1999) 8:586–95. 10.1191/096120399680411317 10568894

[16] Hochberg MC. Updating the American college of rheumatology revised criteria for the classification of systemic lupus erythematosus. *Arthritis Rheum.* (1997) 40:1725. 10.1002/art.1780400928 9324032

[17] Feng X Wang D Chen J Lu L Hua B Li X Inhibition of aberrant circulating Tfh cell proportions by corticosteroids in patients with systemic lupus erythematosus. *PLoS One.* (2012) 7:e51982. 10.1371/journal.pone.0051982 23284839 PMC3524129

[18] Ma CS Deenick EK Batten M Tangye SG. The origins, function, and regulation of T follicular helper cells. *J Exp Med.* (2012) 209:1241–53. 10.1084/jem.20120994 22753927 PMC3405510

[19] Weinstein JS Hernandez SG Craft J. T cells that promote B-cell maturation in systemic autoimmunity. *Immunol Rev.* (2012) 247:160–71. 10.1111/j.1600-065X.2012.01122.x 22500839 PMC3334351

[20] Crotty S. T follicular helper cell differentiation, function, and roles in disease. *Immunity.* (2014) 41:529–42. 10.1016/j.immuni.2014.10.004 25367570 PMC4223692

[21] Yang X Yang J Chu Y Wang J Guan M Zhu X T follicular helper cells mediate expansion of regulatory B cells via IL-21 in lupus-prone MRL/lpr mice. *PLoS One.* (2013) 8:e62855. 10.1371/journal.pone.0062855 23638156 PMC3634758

[22] Le Coz C Joublin A Pasquali JL Korganow AS Dumortier H Monneaux F. Circulating Tfh subset distribution is strongly affected in lupus patients with an active disease. *PLoS One.* (2013) 8:e75319. 10.1371/journal.pone.0075319 24069401 PMC3777901

[23] Kyttaris VC. Systemic lupus erythematosus: from genes to organ damage. *Methods Mol Biol.* (2010) 662:265–83. 10.1007/978-1-60761-800-3_13 20824476 PMC3153363

[24] Mortensen ES Fenton KA Rekvig OP. Lupus nephritis: the central role of nucleosomes revealed. *Am J Pathol.* (2008) 172:275–83. 10.2353/ajpath.2008.070563 18187568 PMC2312358

[25] Su D Liu R Li X Sun L. Possible novel biomarkers of organ involvement in systemic lupus erythematosus. *Clin Rheumatol.* (2014) 33:1025–31. 10.1007/s10067-014-2560-z 24599678

[26] Helve T Teppo AM Kurki P Wegelius O. Circulating DNA-antibodies in systemic lupus erythematosus. *Rheumatol Int.* (1982) 2:103–6. 10.1007/BF00541161 6761831

[27] Elkayam O Burke M Vardinon N Zakut V Ben Yitzhak R Paran D Autoantibodies profile of rheumatoid arthritis patients during treatment with infliximab. *Autoimmunity.* (2005) 38:155–60. 10.1080/08916930400021378 16040336

[28] Simpson N Gatenby PA Wilson A Malik S Fulcher DA Tangye SG Expansion of circulating T cells resembling follicular helper T cells is a fixed phenotype that identifies a subset of severe systemic lupus erythematosus. *Arthritis Rheum.* (2010) 62:234–44. 10.1002/art.25032 20039395

[29] Pallikkuth S Parmigiani A Silva SY George VK Fischl M Pahwa R Impaired peripheral blood T-follicular helper cell function in HIV-infected nonresponders to the 2009 H1N1/09 vaccine. *Blood.* (2012) 120:985–93. 10.1182/blood-2011-12-396648 22692510 PMC3412336

